# Stress specifically deteriorates working memory in peripheral neuropathic pain and fibromyalgia

**DOI:** 10.1093/braincomms/fcad194

**Published:** 2023-07-05

**Authors:** Henrik Børsting Jacobsen, Aurora Brun, Audun Stubhaug, Silje Endresen Reme

**Affiliations:** The Mind Body Lab, Department of Psychology, University of Oslo, Oslo 0373, Norway; Department of Pain Management and Research, Oslo University Hospital, Oslo 0853, Norway; The Mind Body Lab, Department of Psychology, University of Oslo, Oslo 0373, Norway; Department of Pain Management and Research, Oslo University Hospital, Oslo 0853, Norway; The Mind Body Lab, Department of Psychology, University of Oslo, Oslo 0373, Norway; Department of Pain Management and Research, Oslo University Hospital, Oslo 0853, Norway

**Keywords:** peripheral neuropathic pain, fibromyalgia, chronic stress

## Abstract

This study aimed to explore the influence of chronic stress, measured through hair cortisol, on executive functions in individuals with chronic pain. We expected that there would be significant differences in chronic stress and executive functioning between pain patients and healthy controls, as well as between primary and secondary pain classifications. We also hypothesized that hair cortisol concentration was predictive of worse performance on tests of executive functions, controlling for objective and subjective covariates. For this study, 122 participants provided a hair sample (*n* = 40 with fibromyalgia; *n* = 24 with peripheral neuropathic pain; *n* = 58 matched healthy controls). Eighty-four of these participants also completed highly detailed testing of executive functions (*n* = 40 with fibromyalgia; *n* = 24 with peripheral neuropathic pain; *n* = 20 healthy controls). To assess differences in stress levels and executive functions, *t*-tests were used to compare patients with controls as well as fibromyalgia with peripheral neuropathic pain. Then, univariate regressions were used to explore associations between stress and executive functioning in both chronic pain classifications. Any significant univariate associations were carried over to hierarchical multivariate regression models. We found that patients with chronic pain had significantly higher cortisol levels than healthy controls, but all groups showed similar executive functioning. Hierarchical multiple regression analyses disclosed that in a model controlling for age, sex and pain medication usage, hair cortisol levels explained 8% of the variance in spatial working memory strategy in individuals with chronic pain. The overall model explained 24% of the variance in spatial working memory. In a second model using imputed data, including both objective and subjectively reported covariates, hair cortisol levels explained 9% of the variance, and the full model 31% of the variance in spatial working memory performance. Higher levels of cortisol indicated worse performance. In this study, an applied measure of chronic stress, namely hair cortisol, explained a substantial part of the variance on a spatial working memory task. The current results have important implications for understanding and treating cognitive impairments in chronic pain.

## Teaser

Jacobsen *et al*. explored how long-term stress could influence executive function in chronic pain. In individuals with peripheral neuropathic pain or fibromyalgia elevated hair cortisol levels were associated with worse performance on a working memory task, but did not significantly influence other executive function domains.

## Graphical Abstract

**Graphical Abstract:**
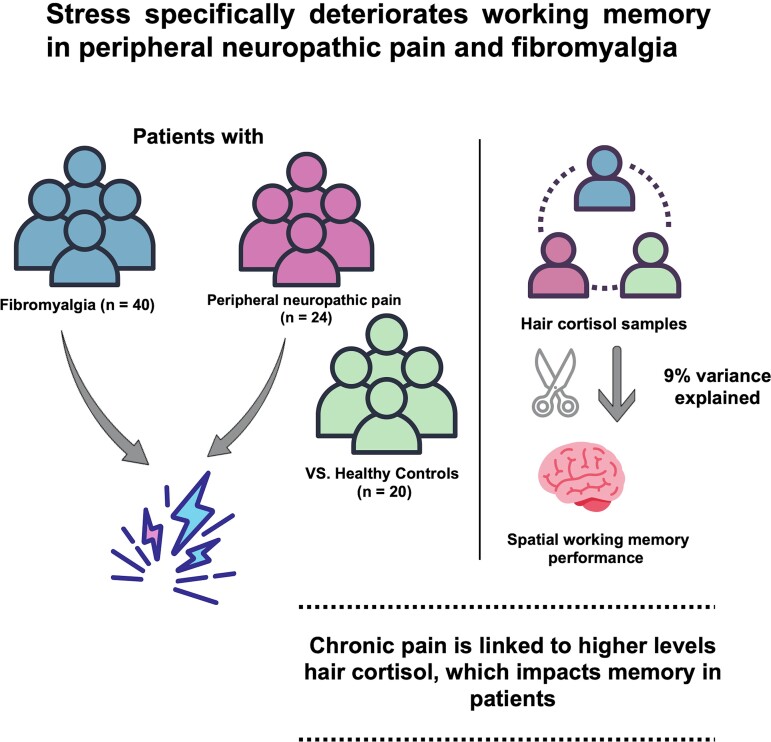

## Introduction

Chronic stress has a debilitating role in chronic pain conditions.^1-3^ While chronic stress may worsen pain over time, less is known about its effects on cognitive capacity. Problems with memory and concentration are frequent and debilitating complaints in these patients, often attributed to the stress of living with chronic pain.^4^

Patients with chronic pain show impairments in inhibitory capacity and response execution, as well as memory updating and retrieval.^4-10^ These impairments are defined within executive functions (EFs),^11^ which enable us to shift attention flexibly, update our working memory, and inhibit pre-potent responses to achieve valued goals.^12^

Suggested explanations for these impairments in chronic pain range from pain intensity to sleep problems, drug use, and chronic stress.^5,6,13,14^ While all these factors may contribute, chronic stress could be particularly salient, given its impact and relevance for cognitive impairments in other conditions.^15,16^

Chronic stress is perhaps best understood as a long-term dysregulation of the Hypothalamic–Pituitary–Adrenal (HPA) axis.^17^ The HPA axis drives its effects through alterations in hormonal regulation, where the hormone cortisol is vital. Increased cortisol exposure over time alters central nervous system activity, affecting both chronic pain^2,3^ and cognitive functions.^18^

A problem for studies of chronic stress is that circulatory assessments of cortisol have low reliability and external validity.^19-21^ Cortisol in hair could thus provide a more reliable approach, as it is a retrospective long-term biomarker of chronic stress.^22^

Four previous studies have assessed hair cortisol concentrations (HCC) in chronic pain. A small pilot study of 14 patients with mixed pain conditions^23^ and a study of 31 patients with endometriosis showed elevated HCC in patients compared to controls, and an association between HCC and pain intensity.^24^ HCC also predicted pain intensity in a sample of 110 individuals with low back pain, but here they did not compare the HCC to people without chronic pain.^25^ Conversely, the last study found no difference in HCC between 20 older women with chronic pain and healthy controls (HCs).^26^

To the best of our knowledge, there have been no studies looking at the association between HCC and cognition in chronic pain. However, HPA axis dysregulation is associated with impairment of cognitive functions in other populations, both healthy adults and clinical samples. Some studies point to an association between elevated HCC levels and impairments in memory and EFs.^15,16,18^ Whereas others do not find an association between HCC and cognitive performance.^27-29^ Conflicting results concerning the association between HCC and cognitive functions indicate, perhaps a nuanced landscape that has not yet been revealed as the research is still limited. From studies of circulatory cortisol, we also know that errors in measurement and timing play a role, and for HCC it is still uncertain to what degree other factors, such as washing, coloring and UV-exposure influence results.

Developing a better understanding of the relationship between chronic stress and EF in chronic pain could both provide insights into the mechanisms of a common complaint as well as guide treatment. We recently showed differences in EF capacity between fibromyalgia (FM) and peripheral neuropathic pain (PNP).^7^ It would therefore be of interest to assess if the impact from stress differs between pain classifications.

The aims of the study were (i) to investigate if pain patients differ from HCs on HCC or EFs; (ii) explore univariate and multivariate associations between HCC and EFs in patients with chronic pain; and (iii) investigate the impact of objective versus subjective data and pain classification.

We hypothesize that patients with chronic pain and HCs will differ on HCC and EFs, and that elevated HCC will predict poorer performance on core EF domains in patients with chronic pain.

## Materials and methods

### Study design and methods

This study was one of three pre-planned analyses stemming from the ‘Brain Fog’ project, where it is described as study number three (ClinicalTrials.gov: NCT02824588, registered 06/07/2016). Pre-registry and the study protocol for the current analyses were published in 2016 and can be found here (grant number: 2016/FO78689).

Data collection took place at the Department of Pain Management and Research, Oslo University Hospital, from July 2016 to February 2020. Patients were recruited from the patient pool of the Department of Pain Management and Research at Oslo University Hospital, Norway. Other pain clinics, patient organizations, and general practitioners were informed about the trial and encouraged to refer patients to the clinic to be considered for inclusion.

### Participants and recruitment

To be considered for inclusion in the study, all participants first met with a specialist in neurology or physical medicine and rehabilitation that assessed if diagnostic criteria for either PNP or FM was applicable. A patient was classified as having PNP if they had peripheral pain and a history of a relevant lesion or disease, which could be assessed by a test, as well as current plausible somatosensory disturbances.^30^ Patients that reported chronic widespread pain, meaning pain in both the left and right side as well as upper and lower parts of the body and pain in the axial skeleton, in addition to at least 11 of 18 tender points were given a diagnosis of FM.^31^ This classification was chosen as it is on par with more recent classification proposals and gave us the opportunity to have a physical examination with the pain physician. It is much more like the rigorous classification given to those who were referred for neuropathic pain. As the potential impact of a thorough examination from an expert doctor on motivation and study results cannot be underestimated, we opted for as close a procedure as possible for the two groups. After the classification as FM pain or PNP was confirmed, patients that also reported problems with memory or concentration were asked to participate in the study. Exclusion criteria were a diagnosis or suspicion of ongoing mania, psychosis, suicidal ideation, scheduled surgery, or pregnancy.

**Figure 1:**
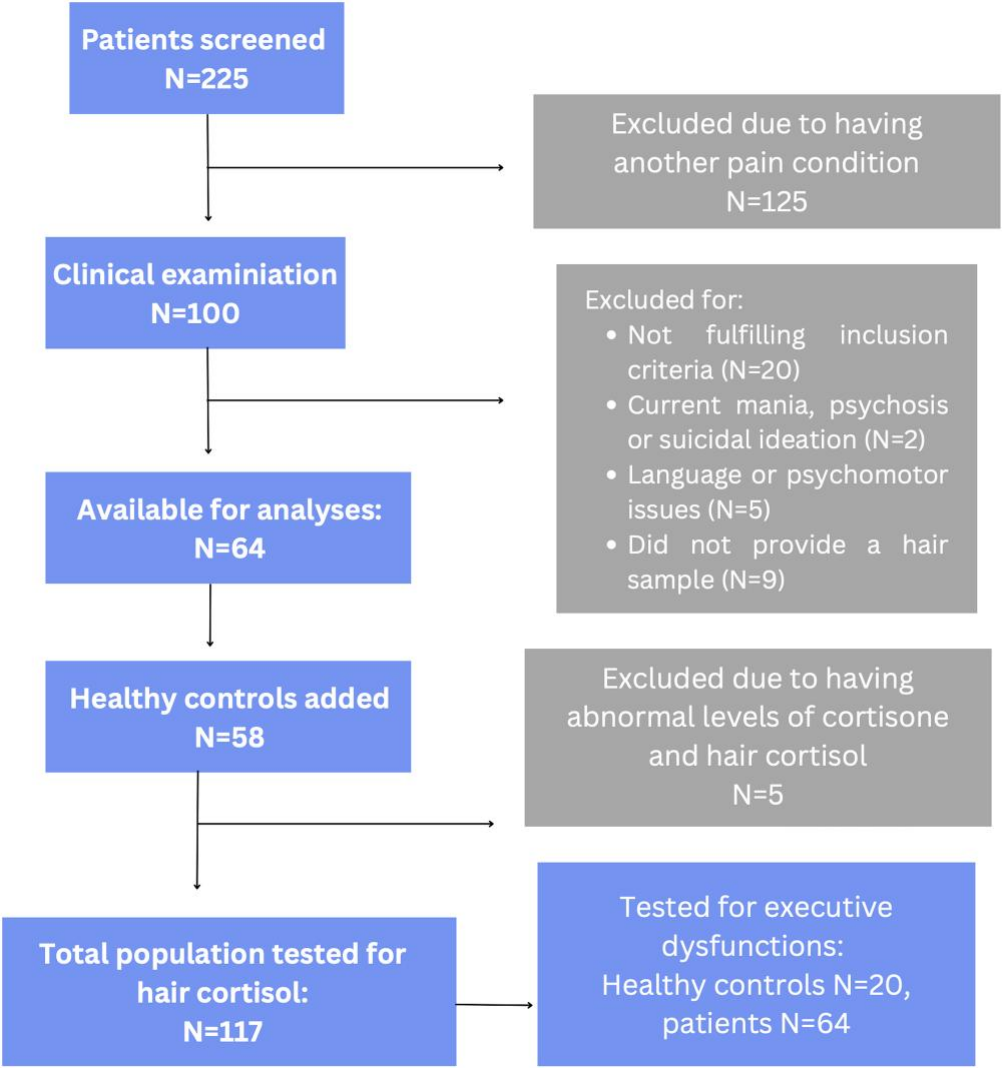
Flow of participants in the study

In addition to the pain patients, 58 HCs matched on age and sex were recruited. All HCs provided informed consent, gave their age, sex, and a hair sample. Twenty of the HCs also completed IQ tests and EF testing to provide us with reference values for EF scores. The process of screening and inclusion of participants is described in Fig. 1.

### Materials

#### Primary outcome: core executive functions

Core EFs were assessed using the Cambridge Automated Neuropsychological Test Battery (CANTAB), which is designed for research purposes. In our protocol and pre-registration, three tests were selected to reflect core EFs: Spatial Working Memory (SWM) for updating, Intra-Extra Dimensional Set Shift (IED) for cognitive flexibility, and the Stop Signal Task (SST) for inhibition and interference control. Moreover, we choose to include the Paired Associates Learning (PAL) test to assess attention-demanding cued recall/visual encoding and retrieval. The measurements chosen as our outcomes were SWM strategy, IED total errors adjusted, SST reaction time last half (SSRT) and PAL total errors. For all tests, a lower score is indicative of better performance. Tests were run on a touchscreen Windows 7 tablet PC. A comprehensive description of each test and outcome can be found elsewhere.^7^

#### Exposure variable: hair cortisol concentration

To measure long-term stress exposure or chronic stress, we analyzed hair samples. All participants provided a sample of at least 3 mm hair cut from the posterior vertex. As one participant did not have hair on the head, a sample of chest hair was included. After sampling, the hair segments were wrapped in aluminum foil and stored at low temperatures in a dry, dark environment. For analysis, samples were sent in batches (patients/volunteers) to the Dresden Lab Service GmbH at the Technische Universität Dresden, Germany.

The analysis followed a standard protocol administered by the Dresden lab, with some changes being made to allow analysis by liquid chromatography-tandem mass spectrometry.^32^ The lower limits of quantification of this assay were below 0.1 pg/mg for cortisol and cortisone. The intraassay coefficients of variance were 10% and 12.5%, respectively.

A recent publication indicated that cortisone levels are central to control for in these analyses due to external agents potentially contaminating the results. We, therefore, chose to use this to detect potential contamination and/or measurement errors.^33^ Participants showing two standard deviations below or above mean values of both cortisone and cortisol were considered contaminated outliers if the reason for this score could not be readily identified (e.g. hydrocortisone treatment). These were then excluded from further analyses (*n* = 5). The sample used for analyses therefore consisted of 64 individuals with chronic pain and 53 HC.

#### Intelligence quotient

Participants completed two subtests, ‘*matrix reasoning*’ and ‘*similarities*’, from The Wechsler Adult Intelligence Scale IV, measuring their non-verbal abstract problem-solving and spatial reasoning abilities, as well as their verbal reasoning abilities and knowledge of concepts. Higher scores are better.

#### Patient reported variables

##### Usual pain intensity

Usual pain intensity was assessed on an 11-point numeric rating scale (NRS), with 0 representing an absence of pain and 10 representing the worst possible pain. This NRS scale has high reliability and validity.^34^

##### Mental distress

Mental distress was measured using the Hopkins Symptom Check List-25 (HSCL-25).^35^ HSCL-25 consists of 25 questions concerning anxiety, depression, and somatization, and we used a validated Norwegian version in the current study.^36^ A mean total score of <1.75 is within the normal range, while a score of 1.75 and above indicates psychological distress and potential need of treatment.^37^

##### Medication use

Participants reported their use of medications upon inclusion. The subsequent journal data was checked against a list of medications provided by the participant’s general practitioner.

##### Sleep problems

Sleep problems were measured with either seven days of actigraphy or a 14-day sleep diary when actigraphy data was incomplete. The data from these measurements were then used to calculate sleep efficiency (SE) by dividing sleep duration (sd) by time spent in bed (tib) (sd/tib = SE).

#### Ethics

An ethics approval was provided by the Regional Committee for Medical Health and Research Ethics in South-Eastern Norway (approval number 2016/595). Data collection was conducted in accordance with The Helsinki Declaration and the ethical principles for Nordic Psychologists.

#### Statistical analysis

Characteristics of the chronic pain sample are described using means ± standard deviations for continuous variables and as numbers and percentages for categorical variables.

Assumptions were checked in the total chronic pain group and separately for each pain classification. For the two pain classifications, outliers or lack of normality was found in one or both groups as assessed by a Shapiro–Wilk’s test (*P* > 0.05) in matrixes, HSCL-25, and sleep efficiency. For the variables that showed heterogeneity of variance, we ran a non-parametric Mann–Whitney *U* test. Age, similarities, pain intensity, and ISI were without outliers, normally distributed, and showed homogeneity of variances. Therefore, a *t*-test was used for these variables.

#### Hypothesis testing

Our first hypothesis required us to test whether HCC or EFs differed between pain patients and HCs. To explore differences between groups, we chose a *t*-test for independent groups to compare means using the raw HCC data and the raw data from EF testing. Following this, we tested to see if there were substantial differences between the two pain classifications, FM and PNP, on HCC using a *t*-test for independent groups.

To test our second hypothesis, we first analyzed univariate associations between HCC and EF scores for all pain patients. As HCC are naturally skewed, the HCC data was log-transformed using a log10 transformation to facilitate the underlying normality assumption of linear regression. Univariate regressions models were then built, testing associations between HCC and the four EF measures.

To control for theoretically derived and suggested covariates besides stress, the next step in analyses was to build multivariate linear regression models to examine if any associations between HCC and EF scores in pain patients remained when controlling for selected covariates. Given the clinical nature of our sample, we chose to examine this in two different multivariate regression models. First, we ran a multivariate model using only objective data provided by testing, registry data and medical journals. This allowed us to control for age, sex, and pain medication. Variables were added in steps to examine explained variance in each step. In the first step, we added age and sex; in the second step, we added pain medications; and in the final third step, we added log10 transformed HCC.

Second, to run a fully adjusted model testing our hypothesis, we added self-reported data from questionnaires in addition to the objective data. We applied the multiple imputation method in SPSS for all self-reported variables with 20% or more missing to not lose participants who had some form of missing. To create a pooled imputed dataset, ten iterations of data generation were used. A total of three imputed variables (pain intensity, sleep efficacy, and mental distress) were used for the final analysis. The distribution of observed and pooled imputed data is shown in the Supplementary material.

In the final hierarchical regression model, we added age and sex in step one; in step two, we added imputed pain intensity; in step three, we added pain medications; in step four, we added imputed sleep efficacy; in step five, we added imputed mental distress; and in step six, we added log-transformed HCC.

As this study was strictly hypothesis-driven, no correction for multiple testing was made, and the threshold for statistical significance was set at *P* < 0.05. All analyses were performed using IBM SPSS software, Version 27.

## Results

Sixty-four individuals with chronic pain were included. They were mainly female (70%, *n* = 46), and in terms of pain conditions, 62.5% (*n* = 40) had FM and 37.5% (*n* = 24) PNP. On average, their pain had lasted for 10.3 years, and the pain intensity in the last week was 6.7 points on the NRS. Approximately half (51.6%) of the participants used opioids, antidepressants, and/or anticonvulsants.

Due to incomplete actigraphy, sleep diaries and technical errors in the online registry, 23% (*n* = 14) of data was missing from the variable pain intensity, 25% (*n* = 16) from HSCL-25% and 34% (*n* = 22) from sleep efficiency (*n* = 22). Table 1 presents an overview of the patient sample, as well as any significant differences between the two pain conditions (PNP and FM).

**Table 1:** Patient characteristics for the total chronic pain sample, pain classifications within the sample and tests of significance between pain classifications

Variables	Total chronic pain sample		Peripheral neuropathic pain		Fibromyalgia		PNP versus FM
	*n* ^a^/M	%/SD	*n*/M	%/SD	*n*/M	%/SD	*P*
Age (years)	46.1	11.4	45.4	12.4	46.6	10.9	0.668
Sex (female)	45	70.3%	15	62.5%	30	75%	0.398
Similarities (WAIS-IV)	22.5	5.2	22.6	4.8	22.5	5.4	0.995
Matrices (WAIS-IV)	17.6	5.1	17.5	5	17.6	5.3	0.872
Taking medication regularly	33	52%	20	83.3%	13	32.5%	0.000^b^
Opioids	12	19%	8	33.3%	4	10%	0.043^b^
Anticonvulsants	17	27%	12	50%	5	12.5%	0.003^b^
Antidepressants	20	31%	10	41.7%	10	25%	0.178
Pain intensity (1–10)	6.7	1.8	6.5	2	6.8	1.7	0.576
HSCL-25 (1–4)	2	0.5	2	0.5	2.1	0.5	0.343
Sleep efficiency (%)	80.3	8.9	80.9	8.4	80	9.3	0.989

a *n* does not equal PNP = 24 or FM = 40 on all variables due to missing data, or participants responding with ‘not applicable’.

b *P* < 0.05.

### Comparing patients and controls on HCC and EF

The 58 HC were attempted to be matched on age and sex, where 88% of the recruited participants were female, and their mean age was 42.2 years. When comparing the samples, the average age in the two groups was comparable, with no significant differences in chronic pain and HC. The Chi-square comparison, however, showed substantially more males in the pain group than in the control group (*P* = 0.02).

Before testing differences in HCC, five hair samples from HC were removed from this analysis because of extreme values indicating measurement error or the use of exogenous hormones such as hydrocortisone, making 53 hair samples available for testing. Independent sample t-tests showed substantial differences in HCC raw scores between pain patients and HCs; however, due to the Levine’s test being significant, the *t*-test results from ‘equal variances not assumed’ were reported here [*t*(67) = 1.98, *P* = 0.05]. Testing for substantial differences in HCC between pain classifications in our sample, an independent *t*-test did not indicate significant differences on HCC between the pain classifications (see Table 2).

**Table 2:** Hair cortisol concentration (pg/mg) in the chronic pain sample and healthy controls

HCC (pg/mg)	Chronic pain sample	Healthy controls	PNP^a^	FM^b^
*n*	64	53	24	40
Mean (SD)	13.7^c^ (33.2)	5.4^c^ (5.4)	18.5 (44.2)	10.9 (24.7)

a PNP = peripheral neuropathic pain.

b FM = fibromyalgia.

c *P* < 0.05.

*T*-tests did not show significant differences between pain patients and a subset of the HCs on measures of EF. Mean outcomes on EF measures are presented in Table 3. Lower scores indicate better performance. Looking only at the raw scores, the subset of HCs did perform better than the chronic pain patients on SWM, SSRT and PAL, but the differences were not statistically significant.

**Table 3:** Core executive functions tested with CANTAB

EF-tests (CANTAB)	Total patient sample (*n* = 64)		Controls (*n* = 20)	
	Mean	SD	Mean	SD
IED total errors	32.2	42.9	32.5	34.8
SWM strategy^a^	32.3	6.8	31.8	5.3
SST reaction time last half (ms)	206.4	39.7	189.6	39.3
PAL total errors 8 shapes	14.8	11.2	12.2	10.9

a The number of times a participant starts a search in a new box on the most difficult stage (remembering as many as six and eight boxes).

### Testing associations between EF and HCC in the chronic pain sample

In initial analyses, univariate regression models showed a single significant relationship between SWM strategy and HCC in chronic pain patients. None of the other three EF measures were significantly associated with HCC (shown in Supplementary material). These were therefore left out of further analyses.

In a hierarchical multivariate regression model using pre-selected covariates with objective data, the covariates explained 15% of the variance in SWM. Sex was the main covariate, with males outperforming females (*P* = 0.002). Including HCClog in the final step of the model explained an additional 8% of the variance (*P* = 0.01). Higher HCClog values were significantly associated with a higher score on SWM in this step, indicating worse performance when controlling for covariates. The overall model predicted SWM [*F*(63) = 4.6, *P* = 0.003] and explained 24% of the variance in SWM performance (see Table 4). This relationship remains when correcting for multiple testing. As we performed three hypothesis tests with an α = 0.05 for each test, a Bonferroni Correction recommended that we use an α = 0.017, still giving a significant association between HCC and SWM.

**Table 4:** Final step (3) in the hierarchical regression model using data provided by objective tests, registries, and medical journals

SWM	*B*	95% CI for *B*		*R* ^2^	Δ*R*^2^
		LL	UL		
Final step				0.24	0.19^c^
Age	0.1	−0.04	0.2		
Sex^a^	−5.5^d^	−8.8	−2.2		
Pain medication^b^	−0.6	−2.4	3.6		
HCC log	4.1^d^	0.9	7.4		

Dependent variable is SWM (Spatial Working Memory Strategy); lower is better.

Model = ‘Enter’ method in SPSS Statistics; *B* = unstandardized regression coefficient; CI = confidence interval; LL = lower limit; UL = upper limit; *R*^2^ = coefficient of determination; Δ*R*^2^ = adjusted *R*^2^.

a Sex: 0 = female, 1 = male.

b Pain medication: 0 = no, 1 = yes.

c *P* < 0.05.

d *P* < 0.01.

A fully adjusted hierarchical regression model was run using a pooled imputed dataset. In this imputed model, covariates explained 22% of the variance in SWM. Sex remained the only significant covariate, with males showing better strategy than females (*P* = 0.002). The final step, including HCClog in the model, explained an additional 9% of the total variance on SWM (*P* = 0.03). Higher HCClog significantly predicted a higher score on SWM, indicating worse performance. The fully adjusted model predicted SWM score [*F* (63) = 3.5, *P* = 0.003] and explained 31% of the variance in SWM performance (see Table 5).

**Table 5:** Final step (6) in the hierarchical regression model using imputed data and SWM strategy as the dependent variable

SWM	*B*	95% CI for *B*		*R* ^2^	Δ*R*^2^
		LL	UL		
Final step				0.31	0.22^c^
Age	0.1	−0.06	0.2		
Sex^a^	−5.5^c^	−10.0	−0.1		
Pain intensity	0.2	−0.7	1.2		
Pain medication^b^	0.7	−2.3	3.7		
Sleep efficacy	0.04	−0.2	0.3		
Mental distress	−1.9	−7.4	3.5		
HCC log10	4.7^c^	0.6	8.8		

Model = ‘Enter’ method in SPSS Statistics; *B* = unstandardized regression coefficient; CI = confidence interval; LL = lower limit; UL = upper limit; *R*^2^ = coefficient of determination; Δ*R*^2^ = adjusted *R*^2^.

a Sex: 0 = female, 1 = male.

b Pain medication: 0 = no, 1 = yes.

c *P* < 0.05.

## Discussion

In this study on chronic stress, chronic pain, and executive functioning, pain patients had substantially higher stress levels than HCs when compared on HCCs. We did, however, not show any significant differences in hair cortisol levels between FM patients and patients with PNP. There were differences, but given the standard deviations and the sample size, these were not significant. Our data indicate that pain classifications are perhaps not as important when evaluating the role of stress in executive dysfunctions related to pain. Somewhat surprising, however, was the finding that pain patients performed nearly as well as HCs on executive functioning.

Although the relationship between pain and decline in executive functioning is frequently reported, it has several weaknesses challenging consistency which could explain the current finding. Even though most studies use validated neuropsychological tests, there are large differences in experimental conditions,^13^ and the definitions and tests of executive functioning tend to vary between studies.^5^ Also a recent review highlighted only selected executive deficits evident in chronic pain, with tests of cognitive flexibility showing no such deficit. Moreover, the review showed a strong interaction between older age and executive dysfunction in the chronic pain cohorts.^10^ This interaction is substantiated by findings on evoked pain in a large population cohort reporting chronic pain.^38^ While surprising, there are several well documented reasons for why our finding could occur in the current sample.

In support of our second hypothesis, we showed that in our sample of pain patients, performance on a SWM task was significantly reduced when cortisol concentrations were high. This relationship withstood controlling for objectively collected data on age, sex, and pain medications, as well as subjectively reported data on pain intensity, sleep problems and mental distress. The final step in our fully adjusted model showed that cortisol concentrations alone accounted for nine percent of the total variance on our SWM task. Our data thus suggests that SWM function might be particularly prone to the negative effects of chronic stress in pain patients.

The finding that pain patients are more chronically stressed than HCs is in line with two previous studies comparing hair cortisol levels with HCs.^23,24^ The unique contribution of this study is our finding that this stress seems to be highly associated with SWM. This processing skill is essential for goal-directed behavior, remembering where an object can be found, and familiarizing oneself with new environments.^39^ Additionally, we frequently use SWM to solve abstract problems through schematic spatial representations.^40^ As SWM is essential for our daily life and work, any reduction in this capacity could have a negative impact on a person living with chronic pain.

To the best of our knowledge, there is only a single previous study investigating how applied measures of chronic stress, such as hair cortisol, can relate to SWM and this supports our current findings. Here, elevated HCCs were found to be predictive of impaired working memory in patients with schizophrenia or bipolar disorders.^41^

Other investigations also support the link between stress and SWM being particularly pronounced for those struggling with chronic pain. A recent study used a single blood sample in FM patients to show that a higher serum cortisol concentration was associated with impairments in SWM.^42^ As in the current study, the observed negative association between cortisol and SWM appeared independent of other cognitive capabilities, self-reported sleep problems and depression.^42^

Although we present only associations, taken together with past studies, we could argue that elevated cortisol levels could drive a specific deterioration in core EFs related to SWM. This appears independent of several other proposed mechanisms such as pain intensity, medication usage, sleep problems, and mental health problems.

Looking at animal studies, we can further speculate as to why such a specific deterioration occurs. SWM capacity is subserved by a network in the brain where the hippocampus and the prefrontal cortex (PFC) are crucial.^43^ In particular, the dorsal hippocampus seems to play a key role in spatial memory.^44^ This is important because this part of the hippocampus is particularly prone to the effects of cortisol as it has a high density of GR receptors.^45^ Changes following chronic stress has been shown to manifest in the hippocampus as remodeling of dendrites in specific areas,^46^ lower neurogenesis,^47^ and turnover of synapses.^48^ Chronic glucocorticoid exposure also eliminates learning-related dendritic spines and disrupts memory retention.^49^

There are also similar results in human studies detailing structures vulnerable to cortisol effects. For instance, it has been shown that the dorsolateral PFC is activated when solving SWM tasks,^50^ and that damage in the right dorsolateral PFC is associated with more errors on an SWM task.^51^ Moreover, a previous investigation in pain patients showed that gray matter volume in the left middle frontal gyrus is positively correlated with SWM.^52^

Taken together, it seems plausible that the negative association between higher cortisol levels and SWM performance we find in our sample is driven by glucocorticoid-induced plasticity in prefrontal and hippocampal areas. However, we have no imaging data to directly support such a claim, and future studies could thus include an imaging component to illuminate this relationship further.

### Strength and limitations

A major strength of this study is that we use a measure of long-term HPA axis activity. HCC reflects a general tendency rather than a momentary snapshot as saliva or plasma cortisol measures do. We have also used objective testing methods for EFs in CANTAB, considered by many as a ‘best practice’ for these types of studies. Self-reported cognitive impairments are often found to relate more to anxiety than actual impairments when rigorously tested. An additional strength is a rigor by which our patients were screened and diagnosed before inclusion in the study.

Nevertheless, several limitations should be accounted for when interpreting our results. The study sample, 64 pain patients and 58 HC, was modest. However, samples of this size, or smaller, are commonly used in studies assessing cognition in pain populations. It could also be conceived as a weakness that we had to impute data in the final model due to missing responses.

### Implications for clinical practice

The current results point to stress regulation as a promising technique for patients suffering from SWM deficits. To improve stress regulation, a person and involved healthcare professionals should first look to lifestyle factors (e.g. sleep, diet, exercise), psychosocial buffers (e.g. appraisal, restructuring, social support), and meaningful activities (hobbies, mind-body practices, nature, etc.).^53^ Additionally, clinical programs and protocols can be used to help the patient relax, focus, and recontextualize, which might alleviate stress. Tools that have already shown great promise are mindfulness and meditation,^54^ hypnosis,^55^ and cognitive behavioral therapy.^56^ Working to make such tools and techniques available to patients easily and at a low cost is important.

## Conclusions

In conclusion, we found that pain patients have substantially higher cortisol levels than controls but perform similarly on EFs. Within our patient sample, those who exhibited a particularly high basal cortisol secretion (high stress-level) performed significantly worse than those with lower levels of cortisol on a test of SWM. No effect of stress (cortisol level) on other EFs was found. This points to stress having a specific influence on SWM rather than generally impairing EFs. It seems plausible that this is because effects cortisol has on the prefrontal and hippocampal structures implied in SWM. The results presented here make a strong, albeit novel case for stress reduction and recuperation being a vital treatment form for patients with pain who struggle with working memory.

## Supplementary Material

fcad194_Supplementary_Data

## Data Availability

The data that support the findings of this study are available on request from the corresponding author. The data are not publicly available due to their containing information that could compromise the privacy of research participants.
