# Supplementary material for: Stress specifically deteriorates working memory in peripheral neuropathic pain and fibromyalgia
Source: Brain Commun. 2023 Jul 5;5(4):fcad194. doi: 10.1093/braincomms/fcad194 (PMC10351603; doi:10.1093/braincomms/fcad194)
Supplement: fcad194_Supplementary_Data [file fcad194_supplementary_data.docx]

**Supplementary table 1 Summary of correlations between HCC, EFs and self-report measures in the pain group**

|  | **1** | **1** | **2** | **2** | **3** | **3** | **4** | **4** | **5** | **5** |
| --- | --- | --- | --- | --- | --- | --- | --- | --- | --- | --- |
|  |  | **(imp.)** |  | **(imp.)** |  | **(imp.)** |  | **(imp.)** |  | **(imp.)** |
| 1. HCC (log) | -- |  |  |  |  |  |  |  |  |  |
| 2. IED total errors (log) | .21 |  | -- |  |  |  |  |  |  |  |
| 3. SWM strategy | .27* |  | .33** |  | -- |  |  |  |  |  |
| 4. SST reaction time last half | .19 |  | .37** |  | .22 |  | -- |  |  |  |
| 5. PAL errors 8 shapes | .11 |  | .12 |  | .11 |  | .02 |  | -- |  |
| 6. Similarities | -.01 |  | -.22 |  | -.43** |  | -.22 |  | -.27* |  |
| 7. Matrices | -.12 |  | -.30* |  | -.30* |  | -.19 |  | -.24 |  |
| 8. Age | .11 |  | .17 |  | .18 |  | .27* |  | .17 |  |
| 9. Sex | .10 |  | -.06 |  | -.34** |  | .03 |  | .05 |  |
| 10. Pain classification | -.03 |  | -.10 |  | .13 |  | .16 |  | -.08 |  |
| 11. Years lived with pain | -.06 |  | -.17 |  | .09 |  | -.01 |  | .00 |  |
| 12. HSCL-25 | .33* | -.27* | -.09 | .05 | .07 | .04 | -.16 | -.13 | .16 | .15 |
| 13. ISI | .32* | .28* | .27 | .15 | .21 | .13 | .00 | -.01 | .35* | .29* |
| 14. Sleep efficiency | .04 | .04 | .12 | .13 | .19 | .14 | .-08 | -.12 | -.17 | -.13 |
| 15. Pain intensity | .20 | .16 | .21 | .17 | .27 | .22 | .17 | .15 | .20 | .14 |
| 16. On meds | -.06 |  | .02 |  | .03 |  | -.03 |  | .08 |  |
| 17. Anticonvulsants | -.03 |  | -.05 |  | -.29* |  | -.07 |  | .02 |  |
| 18. Opioids | .01 |  | .30* |  | .03 |  | .15 |  | -.03 |  |
| 19. Antidepressants | -.09 |  | .01 |  | .07 |  | .04 |  | -.05 |  |

Pearson’s product-moment correlations were used for continuous variables and point-biserial correlations for a combination of continuous and dichotomous variables.

**P* < 0.05.

***P* < 0.01

**Supplementary table 2 Missing data: observed and imputed data for pain patients**

| **Variable** | **% Missing** | **Observed** | | **Imputed** | |
| --- | --- | --- | --- | --- | --- |
|  |  | ***M/n*** | ***SD/%*** | ***M/n*** | ***SD/%*** |
| Age (years) | 0 | 46.12 | 11.41 | NA^a^ | NA |
| Sex (female) | 0 | 45 | 70.3% | NA | NA |
| Education | 25 | NA | NA | NA | NA |
| Similarities (WAIS-IV) | 10.9 | 22.5 | 5.17 | NA | NA |
| Matrices (WAIS-IV) | 3.1 | 17.55 | 5.11 | NA | NA |
| Taking medication regularly | 0 | 33 | 51.6% | NA | NA |
| Years lived with pain | 14.1 | 10.29 | 11.31 | NA | NA |
| Pain intensity last week (0-10) | 23.4 | 6.65 | 1.76 | 6.65 | 1.56 |
| HSCL-25 (1-4) | 25 | 2.02 | 0.45 | 2.05 | 0.40 |
| ISI (0-28) | 28.1 | 13.98 | 6.77 | 14.41 | 5.98 |
| Sleep efficiency (%) | 34.4 | 80.3 | 8.93 | 79.59 | 7.72 |

^a^NA = Not Applicable
